# A new species of *Camchaya* (Asteraceae, Vernonieae) from Thailand

**DOI:** 10.3897/phytokeys.12.3221

**Published:** 2012-05-08

**Authors:** Sukhonthip Bunwong, Pranom Chantaranothai, Sterling C. Keeley

**Affiliations:** 1 Maejo University Phrae Campus, Mae Sai, Rong Kwang, Phrae 54140, Thailand; 2 Applied Taxonomic Research Center, Department of Biology, Faculty of Science, Khon Kaen University, Khon Kaen 40002, Thailand; 3 Department of Botany, University of Hawaii at Manoa, 3190 Maile Way, Honolulu, HI 96822, USA

**Keywords:** Asteraceae, Compositae, Vernonieae, *Camchaya*, Thailand, new species

## Abstract

*Camchaya thailandica* Bunwong, Chantar. & S.C.Keeley, **sp. nov.** from Phu Phrabat Historical Park, Udon Thani, Thailandis described as a new species. Plant of this new species are similar to *Camchaya gracilis* (Gagnep.) Bunwong & H.Rob. but differ in having ovate phyllaries without margin spines, 10-ribbed achenes, and broadly ovate leaves. This species is a rare endemic known only from the type collection and probably confined to open areas of sandstone hills in Udon Thani province.

## Introduction

Koyama (1984) described six species of *Camchaya* in Thailand, recognizing the genus based on the presence of 5–10 ribbed achenes and a bristle-like pappus on some florets, thus broadening earlier concepts of the genus by Kerr (1935) and Kitamura (1968).  Bunwong et al. (2009) reviewed the status of *Camchaya eberhardtii* (Gagnep.) Kitam., *Iodocephalus glandulosus* Kerr and *Iodocephalus eberhardtii* Gagnep. and recognized these taxa as *Iodocephalopsis eberhardtii* (Gagnep.) Bunwong & H.Rob. while *Iodocephalopsis gracilis* Thorel ex Gagnep. was recognized as *Camchaya gracilis* (Gagnep.) Bunwong & H.Rob. Continuing with these studies Bunwong (2010) found seven species of *Camchaya* including a new taxon, *Camchaya thailandica*, described here. *Camchaya thailandica* is one of the five endemic Thai species: including *Camchaya pentagona*, *Camchaya spinulifera*, *Camchaya tenuiflora*, and *Camchaya gracilis*. The two remaining species, *Camchaya kampotensis* and *Camchaya loloana* extend beyond Thailand into Laos, Cambodia and adjacent Yunnan, China.

## Methodology

*Camchaya* specimens examined were obtained from the following herbaria: AAU, BK, BKF, BM, CMU, E, K, KKU, L, P, QBG, and US. All measurements given herein were taken from field notes, dried herbarium specimens, and spirit collections. Pollen and achenes were obtained from field collections around Thailand by the first author. Pollen samples for the SEM work were acetolyzed (Erdtman 1960). Acetolyzed pollen was freeze-dried using the critical point drying method. Acetolyzed pollen, unacetolyzed achenes and leaf surfaces were then placed on specimen stubs with double sided silver tape and sputter coated with gold. Photomicrographs were taken with SEM (LEO, 1450VP; Applied Taxonomic Research Center, Department of Biology, Faculty of Science, Khon Kaen University).

## Taxonomy

### Camchaya thailandica

Bunwong, Chantar. & S.C.Keeley sp. nov.

urn:lsid:ipni.org:names:77119225-1

http://species-id.net/wiki/Camchaya_thailandica

#### Type.

**Figure 1.**
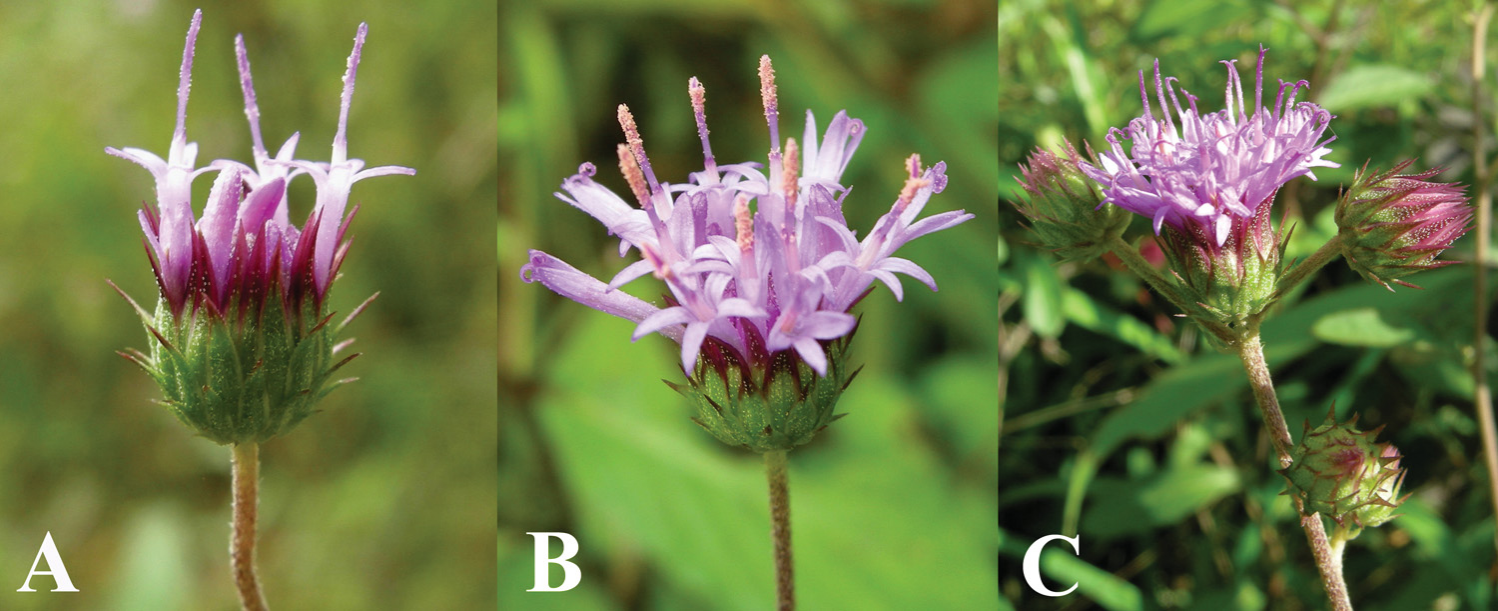
Capitulescences, **A–B** terminal **C** axillary. Note that phyllaries are in 5–6 series and without margin spines.

**Figure 2.**
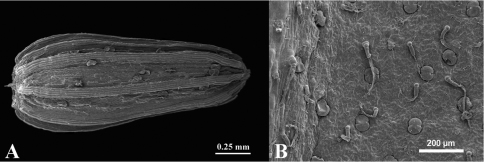
SEM micrographs, **A** Achene with glands and 10 ribs, **B** Abaxial leaf surface with cylindrical hairs and capitate glands.

**Figure 3.**
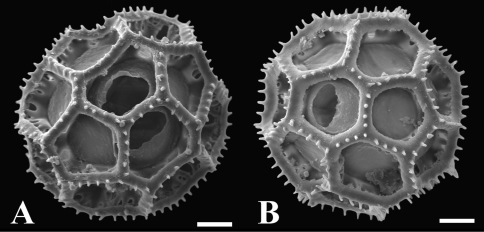
**A–B** SEM micrographs of acetolysed pollen show 6-porate echinolophate pollen. (scale bars = 6 µm).

Thailand. Prov. Udon Thani, rare on rocky areas in Phu Phrabat Historical Park. alt. 300 m, 17°43.84'N; 102°29.65'E, 29 September 2007 (flower) S. Bunwong 328 (holotype KKU, isotype US) (Figures 1–3). Known only from the type collection.

Annual. Inflorescences axillary or terminal, pedunculate. Phyllaries imbricate in 5–6 series, arachnoid-glandular, apices purple. Differs from *Camchaya gracilis* (Gagnep.) Bunwong & H.Rob. in having ovate acuminate rather than broadly ovate acute phyllaries without margin spines, 10-ribbed rather than 4–5-ribbed achenes, and broadly ovate leaves.

Annual herbs, 50–100 cm tall. *Stems* erect, rounded, inconspicuously ribbed, scabrous, hairs uniseriate, T-shaped, and glandular. *Leaves* alternate; petioles to 2 cm long; blades elliptic to oblong, 3–8 by 2–3 cm, chartaceous; bases attenuate, margins serrate, apices acute; both surfaces pubescent, hairs cylindric, T-shaped, and glandular, lateral veins 5–10 pairs. *Capitulescences* terminal and axillary, corymbose. *Capitula* pedunculate, involucres broadly campanulate, 5–6 mm diam. *Receptacles* convex, 2.5–3 mm diam., glabrous. *Phyllaries* 5–6-seriate, imbricate,light green with purple apices, 7–8 mm long, margins pale, without margin spines, outer surfaces arachnoid-glandular; the outer and the middle ones ovate, apices acuminate; the inner ones lanceolate to oblong, apices acuminate. *Florets* 50–70; corollas infundibular, purple, pubescent, glands capitate; tubes 6–7 mm long; lobes 2.5–3 mm long. *Anthers ca*. 2 mm long, bases rounded, apical appendages acute. *Styles* purple, 6–7 mm long; branches 2–2.5 mm long; sweeping hairs on the outer surfaces reaching below style bifurcation. *Achenes* obovate, *ca*. 1.5 mm long, glandular, 10-ribbed, carpopodium absent. *Pappus* bristles, uniseriate, 1–2 mm long, sometimes absent, deciduous. *Pollen* echinolophate, 6-porate, without micropuncta.

#### Distribution.

Endemic to Thailand. Only found in Phu Phrabat Historical Park, Udon Thani province.

#### Ecology.

Rocky areas of sandstone hills, scattering in dipterocarp forest, flowering from November and December.

#### Discussion.

*Camchaya thailandica* is similar to *Camchaya gracilis* in having ovate phyllaries without spines on the margin, but differs in having 10-ribbed achenes and a broadly ovate leaf shape. Its 6-porate echinolophate pollen is unique to *Camchaya* (Bunwong and Chantaranothai 2008) and places it firmly in this genus. Additionally, this species has an inconspicuous carpopodium which is common in *Camchaya*.

##### Key to the genus *Camchaya*

**Table:** 

1	Phyllaries broadly ovate without margin spines	2
–	Phyllaries broadly ovate with margin spines	3
2	Achenes 4–5-ribbed	*Camchaya gracilis*
–	Achenes 10-ribbed	*Camchaya thailandica*
3	Achenes 5 (6–9)-ribbed	*Camchaya pentagona*
–	Achenes 10-ribbed	4
4	Phyllaries eglandular, margin spines to 10 mm long	*Camchaya spinulifera*
–	Phyllaries glandular, margin spines to 5 mm long	5
5	Phyllaries acuminate; achenes 2.5–3 mm long	*Camchaya kampotensis*
–	Phyllaries aristate or apiculate; achenes 1.5–2 mm long	6
6	Leaves with T-shaped hairs; phyllaries spinose ≤ 1 mm long	*Camchaya loloana*
–	Leaves without T-shaped hairs; phyllaries spinose ≥ 1 mm long	*Camchaya tenuiflora*

## Supplementary Material

XML Treatment for Camchaya thailandica
